# The Dimensions of Primary Mitochondrial Disorders

**DOI:** 10.3389/fcell.2020.600079

**Published:** 2020-11-26

**Authors:** Lea D. Schlieben, Holger Prokisch

**Affiliations:** ^1^School of Medicine, Institute of Human Genetics, Technical University of Munich, Munich, Germany; ^2^Institute of Neurogenomics, Helmholtz Zentrum München, Munich, Germany

**Keywords:** mitochondrial disease, genetics, mutation, diagnostic, challenges

## Abstract

The concept of a mitochondrial disorder was initially described in 1962, in a patient with altered energy metabolism. Over time, mitochondrial energy metabolism has been discovered to be influenced by a vast number of proteins with a multitude of functional roles. Amongst these, defective oxidative phosphorylation arose as the hallmark of mitochondrial disorders. In the premolecular era, the diagnosis of mitochondrial disease was dependent on biochemical criteria, with inherent limitations such as tissue availability and specificity, preanalytical and analytical artifacts, and secondary effects. With the identification of the first mitochondrial disease-causing mutations, the genetic complexity of mitochondrial disorders began to unravel. Mitochondrial dysfunctions can be caused by pathogenic variants in genes encoded by the mitochondrial DNA or the nuclear DNA, and can display heterogenous phenotypic manifestations. The application of next generation sequencing methodologies in diagnostics is proving to be pivotal in finding the molecular diagnosis and has been instrumental in the discovery of a growing list of novel mitochondrial disease genes. In the molecular era, the diagnosis of a mitochondrial disorder, suspected on clinical grounds, is increasingly based on variant detection and associated statistical support, while invasive biopsies and biochemical assays are conducted to an ever-decreasing extent. At present, there is no uniform biochemical or molecular definition for the designation of a disease as a “mitochondrial disorder”. Such designation is currently dependent on the criteria applied, which may encompass clinical, genetic, biochemical, functional, and/or mitochondrial protein localization criteria. Given this variation, numerous gene lists emerge, ranging from 270 to over 400 proposed mitochondrial disease genes. Herein we provide an overview of the mitochondrial disease associated genes and their accompanying challenges.

## Introduction

Mitochondria are dynamic ubiquitous organelles with innumerable functions in various cellular and metabolic pathways. Energy production via the oxidative phosphorylation (OXPHOS) represents the most prominent function of mitochondria (Duchen, 2004). Consisting of five protein complexes and two electron carriers embedded in the inner mitochondrial membrane, the OXPHOS generates ∼ 90% of the body’s energy. An electrochemical gradient across the inner mitochondrial membrane is the driving force of adenosine triphosphate (ATP) synthesis by the fifth OXPHOS complex (ATP synthetase) (Papa et al., 2012). Mitochondria also have a decisive contribution to other cellular processes, such as initiation of apoptotic cell death, calcium homeostasis, heme and iron-sulfur cluster biosynthesis, and amino acid and lipid metabolism (Nunnari and Suomalainen, 2012; Gorman et al., 2016).

Nucleated cells of multicellular eukaryotes contain hundreds to thousands of mitochondria, with numbers varying among different tissues depending on energy demand (Cole, 2016). Uniquely, mitochondria have retained their own DNA, the mitochondrial DNA (mtDNA), a circular, double-stranded genome consisting of 37 genes, with each mitochondrion containing 2–10 copies of mtDNA (Anderson et al., 1981; Keogh and Chinnery, 2015). The 37 genes in total encode 13 proteins involved in OXPHOS, 22 mitochondrial tRNAs, and two subunits of the mitochondrial ribosomes. The additionally required ∼ 1500 proteins of the mitochondrial proteome are encoded by the nuclear genome and actively imported into the mitochondria (Pagliarini et al., 2008; Calvo et al., 2016; Alston et al., 2017). Unlike the nuclear DNA (nDNA), which displays Mendelian inheritance patterns, inheritance of mtDNA is exclusively maternal while paternal mtDNA is degraded after fertilization (Al Rawi et al., 2011).

## Mitochondrial Disorders

The term “mitochondrial disorder” refers to a large group of genetically defined disorders, leading to direct defects of the pyruvate root of the OXPHOS (DiMauro and Garone, 2010; Mancuso et al., 2017; Stenton and Prokisch, 2020). The clinical diagnosis of individuals with mitochondrial disease poses a major challenge to physicians. Considering the presence of mitochondria in all nucleated cells, clinical symptoms can selectively involve only a single organ or be multisystemic affecting predominantly, but not exclusively, organs with the highest energy requirements, such as the heart, brain, and skeletal muscles. Moreover, the age of disease presentation can vary substantially. Clinical features are manifold and often overlap with other systemic or neurological diseases (Ng and Turnbull, 2016). Many common clinical symptoms, such as short stature, migraine, diabetes mellitus, and blindness, are not specific to mitochondrial disorders, and are also prevalent in the population. They may therefore present due to the primary genetic defect or as an unrelated clinical entity. Only a fraction of individuals display a unique combination of clinical manifestations belonging to a distinct clinical syndrome described more frequently, such as mitochondrial encephalopathy, lactic acidosis and stroke-like episodes (MELAS), chronic progressive external ophthalmoplegia (CPEO), maternally inherited diabetes and deafness (MIDD), and Leber’s hereditary optic neuropathy (LHON). The clinical course and mortality rates of affected individuals vary widely owing to the heterogenous disease manifestations, with pediatric disease onset often being multisystemic and rapidly progressive in association with high mortality rates (Gorman et al., 2016).

The concept of mitochondrial disorders was initially described in Luft et al. (1962) on the basis of clinical, biochemical, and morphological evidence. In this premolecular era, mitochondriopathies were grouped into general biochemical pathways, such as defects in the substrate transport, tricarboxylic acid (TCA) cycle, or the pyruvate dehydrogenase complex. The term “mitochondrial disorder” has been employed for describing defects in the mitochondrial OXPHOS (DiMauro and Garone, 2010). At present, however, no consensus on uniform and standardized biochemical criteria for the definition of mitochondriopathies exists. Mitochondrial diseases are suspected on clinical grounds, but muscle biopsies and biochemical markers are necessary to validate the suspicion (Koenig, 2008; DiMauro, 2011). Biochemical screening methods for mitochondrial biomarkers in blood, urine, and spinal fluid, typically include the measurement of pyruvate and lactate, amino acids, urine organic acids, and plasma acylcarnitines. More recently, biochemical screenings also include new biomarkers for mitochondrial diseases, such as FGF-21 and GDF-15 (Suomalainen et al., 2011; Parikh et al., 2015; Montero et al., 2016). All of these biochemical measurements can be indicators for the presence of a mitochondriopathy, however, given the suboptimal specificity and sensitivity of these biomarkers, the confirmation of a specific diagnosis is challenging. In addition, no uniform or standardized guidelines for the biochemical evaluation of a suspected disease exist and multiple biochemical assays are conducted in several laboratories, ranging from single enzyme measures to metabolic flow or ATP production measurements in fresh or frozen tissues processed following various protocols (Haas et al., 2008). Given their post-mitotic properties, skeletal muscle biopsies are the tissue of choice and are the most commonly sampled in biochemical analyses (Phadke, 2017). Normal biochemical findings do not rule out an OXPHOS deficiency in other tissues or a defect not examined in the artificial system, such as a cofactor malfunction, if the cofactor was supplemented in the assay. At the same time, it has become apparent that in some patients the significantly reduced activity in the conducted assay is not always attributable to a defect in the OXPHOS enzymes, but to secondary mitochondrial dysfunctions or artifacts of the biochemical measurements. The secondary involvement of mitochondria in diseases is thus not considered as primary mitochondriopathy (Mancuso et al., 2017). Disorders contributing to secondary mitochondrial dysfunctions are being detected with an increasing frequency, making the diagnosis of mitochondrial disorders based on biochemical findings in tissues evermore challenging (Niyazov et al., 2016; Parikh et al., 2019).

The molecular era of mitochondrial medicine began in 1988 with the description of the first disease-causing mutation in the mtDNA (Holt et al., 1988; Wallace et al., 1988; Zeviani et al., 1988). Since then, the identification of pathogenic variants has become the essential key for the molecular genetic confirmation of clinically suspected mitochondrial disorders and genetic definition of mitochondriopathies has subsequently gained increasing relevance over biochemical definition. Due to the mtDNA encoding only OXPHOS proteins and additional genetic elements required for mtDNA replication, transcription and translation of those peptides, an undisputed fact was that diseases associated with pathogenic variants of the mtDNA are mitochondrial disorders. Primary mitochondrial disorders are, however, also caused by pathogenic variants in nDNA encoded genes functionally relevant for the mitochondrial OXPHOS. The dual genetic control of mitochondria means that mutations can follow all possible modes of inheritance: maternal, autosomal-recessive, autosomal-dominant, and X-linked. For both, nDNA and mtDNA, pathogenic variants can occur *de novo* (DiMauro et al., 2004).

Although mutations in mitochondrial disease genes are individually rare, mitochondrial disorders represent the largest group of inborn errors of metabolism, with a collective lifetime risk of 1.6 in 5,000 (Ferreira et al., 2019; Tan et al., 2020). To date, pathogenic variants in more than 400 genes, of both mitochondrial and nuclear origin, have been ascribed as causes of mitochondriopathies (Frazier et al., 2019; Falk, 2020; Rahman, 2020; Stenton and Prokisch, 2020).

In patients with mtDNA encoded disease, inheritance and clinical presentation are further complicated by the presence of multiple mtDNA copies, both wild-type and mutant, in each cell (Larsson and Clayton, 1995). The level of this so called “heteroplasmy” often needs to exceed a tissue specific threshold to cause a biochemical defect and an associated mitochondrial disorder. Such threshold levels have been shown to vary widely between different tissues and mtDNA mutations. The level of heteroplasmy is not only associated with variable clinical manifestations but also disease severity (Khan et al., 2015; Grady et al., 2018). Insufficient genotype-phenotype correlation, even within defined clinical syndromes, are additional complications in the genetics of mitochondrial diseases (French et al., 2019). Leigh syndrome, a progressive neurodegenerative disorder in childhood, exemplifies the genetic heterogeneity of mitochondriopathies as it is associated with defects in more than 90 different genes (Rahman et al., 2017). Conversely, the straightforward association of a single gene with a defined clinical presentation and mode of inheritance is uncommon in mitochondrial diseases. A classic example displaying the genetic pleiotropy of mitochondrial disorders is the most common disease-causing mtDNA mutation m.3243A > G in *MT-TL1* causing a wide range of phenotypes, including MELAS, CPEO, and MIDD (Nesbitt et al., 2013).

This immense clinical and genetic heterogeneity of mitochondrial disorders, in conjunction with inadequate genotype-phenotype correlations, makes the reliable diagnosis of patients a demanding task, requiring extensive expertise of clinicians from all specialties. Many patients thus undergo a diagnostic odyssey with a multitude of consultations, conflicting diagnoses, and repeated, often invasive tests (Grier et al., 2018).

Although remarkable progress has been made in the field of mitochondrial medicine, the complexity of mitochondrial diseases continues to add to a shortage of therapeutic options for patients. The incomplete understanding of disease pathomechanisms, the rareness of the individual diseases, the inaccessibility of the double-membraned mitochondrion for a number of drugs, as well as the small number of biomarkers and outcome measures to demonstrate treatment effects, have contributed to a lack of licensed curative therapies for patients. Many early attempts at therapy development have sought to improve the function of the respiratory chain, by administering cofactor supplements such as carnitine, niacin, and thiamine (Pfeffer et al., 2013). With the exception of a few of these deficiencies in the biosynthesis or transport of cofactors and vitamins (Koch et al., 2017), the majority of patients still receive only symptomatic and supportive treatments, such as exercise, hearing aids, or reduction of toxic metabolites (Distelmaier et al., 2017; Hirano et al., 2018; Pitceathly et al., 2020). Thanks to technological advances, however, the knowledge of mitochondrial diseases is improving. This knowledge, coupled with the collection of larger cohorts of patients in to registries, enables an increasing number of clinical trials to be conducted, with many more expected in the next years.

## Developments in the Molecular Diagnostics of Mitochondrial Disorders

The molecular era of mitochondrial medicine began in 1988 with the discovery of the first pathogenic variants causing mitochondrial disease. Within 1 year large-scale deletions in the mtDNA causing mitochondrial myopathy (MIM 251900) or Kearns-Sayre syndrome (KSS, MIM 530000) shortly followed by an mtDNA point mutation in *MT-ND4* causing LHON (MIM 5350000), were identified (Holt et al., 1988; Wallace et al., 1988; Zeviani et al., 1988). With the discovery of pathogenic variants in the mitochondrial genome, the suspicion of a mitochondrial disorder based on clinical and biochemical markers could be unambiguously confirmed. Given the small size of the mitochondrial genome, the last decade of the first millennium in mitochondrial research was dominated by discoveries of pathogenic variants in the mtDNA (DiMauro and Garone, 2010). Mitochondrial disease associated mutations have now been reported for almost all 37 mtDNA genes. The only exception is *MT-RNR2*, for which proof of pathogenicity has not yet been sufficiently validated (Lott et al., 2013). Mutations in the mtDNA, however, could not explain the entire heritability of mitochondrial disorders and shortly after the identification of the first pathogenic mtDNA variants as genetic causes of mitochondrial diseases, the first nuclear encoded cause, defects in the X-chromosomal encoded gene *PDHA1*, was reported (Endo et al., 1989). Already, this first nuclear encoded defect exceeded the strict biochemical concept of impaired OXPHOS for mitochondrial diseases by a dysfunction in the PDH. However, the classification of PDH deficiencies as mitochondrial diseases is undisputed.

The identification of pathogenic variants in the mtDNA and nDNA guided the beginning of the genetic era of diagnostics in suspected mitochondrial disease. Traditionally, molecular diagnoses of suspected mitochondrial disorders relied upon clinical phenotyping and blood, urine, and skeletal muscle tests evaluating the biochemical evidence of mitochondrial defects. Biochemical screening studies then guided targeted candidate gene sequencing of patient cohorts with similarities in the clinical and biochemical phenotype. The limited association of single genes with recognizable clinical presentations, in combination with the large number of mitochondrial disease genes, often made the sequencing of individual genes a laborious and time-consuming approach. Hence, candidate gene sequencing is effective only for clear clinical phenotypes explained by a low number of distinct pathogenic variants, due to the technique being sensitive enough for variant detection and segregation analysis.

In the last decade, the restricted analysis of the genome by conventional candidate gene sequencing methods was overcome with the advent of whole-exome sequencing (WES) and whole-genome sequencing (WGS), both serving to study the genetic background of this disease group (Haack et al., 2012). Advances in the next generation sequencing (NGS) technology have remarkably reduced the cost and workload of sequencing and laboratory protocols, allowing the implementation of these methods into routine diagnostics at many centers, and the analysis of large patient cohorts (Stenton and Prokisch, 2020; Wetterstrand, 2020). Capturing data on all genes allows us to identify not only known pathogenic variants, but also other outcomes including: (1) novel variants in mitochondrial disease genes, (2) suspected pathogenic variants in candidate genes, (3) variants in disease genes not associated with mitochondrial disease yet with a similar phenotypic presentation, and (4) a number of variants of uncertain significance (VUS). Thereby, WES and WGS do not only help to distinguish mitochondrial from non-mitochondrial disorders presenting clinically as mitochondriopathy, but also help to identify mitochondrial diseases in patients not suspected of having mitochondrial dysfunction. As the number of examined genes increases, more rare variants can be discovered, requiring interpretation of their clinical relevance.

Since the detection of the first mitochondrial disease gene *ACAD9* by NGS in 2010, the number of reported mitochondrial disease genes has duplicated, exemplifying the technology’s diagnostic power (Haack et al., 2010). In the pre-NGS era approximately five mitochondrial disease genes were reported annually, increasing to over 15 genes annually after the advent of NGS methods in 2010 (Stenton and Prokisch, 2020). Application of WES and WGS in patients with suspected mitochondrial disease for whom extensive prior clinical analysis had failed to return a diagnosis achieves diagnostic yields of approximately 50% (Taylor et al., 2014; Wortmann et al., 2015; Kohda et al., 2016; Pronicka et al., 2016). The success of unbiased NGS methods results in the less frequent analysis of invasive skeletal muscle biopsies. By reducing the frequency of biochemical examinations, the classic definition of a mitochondrial disease is further neglected leading to the argument for utilization of genetic definitions (Wortmann et al., 2017). In line with the concept that mtDNA mutations cause an OXPHOS disease, mutations in nuclear genes with products considered functionally relevant for the OXPHOS, covering mtDNA replication and transcription, mitochondrial translation and pyruvate oxidation root, are classified as causes of mitochondrial diseases, not always associated with an OXPHOS deficiency in all patients.

Increasing integration of WES and WGS into routine diagnostics of mitochondrial diseases improved diagnostic rates remarkably. Nevertheless, a significant number of patients with suspected mitochondrial disease do not receive a genetic diagnosis due to challenges in variant detection and interpretation. Other methods contribute to closing the diagnostic gap further.

For the interpretation of VUS new tools are becoming useful in the diagnosis of Mendelian diseases. Quantification of the gene products can help to interpret unclear pathogenic variants in case they result in reduced levels of proteins, making proteomics a powerful tool to complement the genetic analysis. Moreover, recent studies demonstrate that whole transcriptome sequencing (RNA-seq) increases the power and sensitivity of detection and interpretation of the large number of VUS identified by DNA sequencing. RNA-seq provides insight into the transcripts of a tissue at a specific time point and is an additional tool following exome sequencing to analyze unsolved cases by high-throughput functional characterization of variants. Of rare disease patients remaining undiagnosed by DNA analysis, by providing evidence for the pathogenicity of variants, RNA-seq allows an additional 17.5% of investigated patients to reach a genetic diagnosis (Cummings et al., 2016; Kremer et al., 2017; Frésard et al., 2019; Gonorazky et al., 2019; Lee et al., 2020).

Despite the potential of such multi-omic approaches to further increase the diagnostic yield, the most commonly used method in current practice for evaluating the pathogenicity of VUS and the impact on OXPHOS remains to be the biochemical examination of tissue biopsies, though we expect this to change in the future.

Taken together, despite the major technological advances in diagnostic tools for rare diseases, the lack of genotype-phenotype correlations, the genetic heterogeneity and the presence of VUS in patients with mitochondrial disease imply that the confirmation of a specific diagnosis often remains a challenge.

## Definition of Mitochondrial Disease Genes

With the increased utilization of sequencing technologies, it is now possible to provide a molecular diagnosis to a large fraction of patients with mitochondrial disease. As a result, mitochondriopathies are meanwhile frequently defined by their genetic cause rather than by biochemical deficiencies.

The number of nuclear genes associated with mitochondrial function is estimated to be ∼1500 (Calvo et al., 2016). So far, 376 of these genes have been associated with human mitochondrial disorders in total and as the routine use of NGS technologies grows, this number is expected to continuously increase (Frazier et al., 2019; Falk, 2020; Rahman, 2020; Stenton and Prokisch, 2020). Due to the capacity to detect pathogenic variants in genes so far not linked to mitochondrial disorders, the criteria for which genes should be classified as associated with a mitochondriopathy are now the subject to discussion.

**FIGURE 1:**
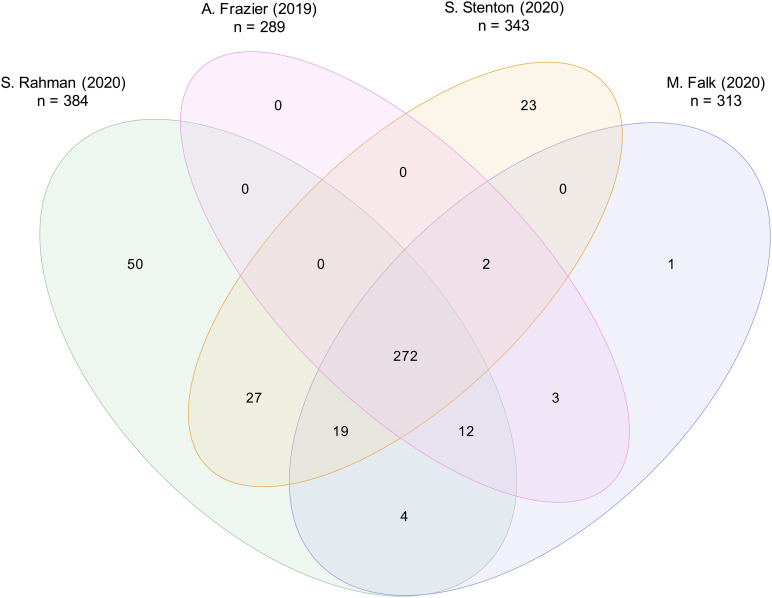
Comparison of four distinct mitochondrial disease gene lists. Mitochondrial disease gene lists of (1) Frazier et al. (2019), listing 289 genes, (2) Rahman (2020), 384 genes, (3) Stenton and Prokisch (2020), 343 genes, and (4) Falk (2020), 313 genes, were compared. A total of 413 distinct genes was reported. A core of 272 mitochondrial disease genes was listed by all four authors.

Considering the mitochondrial disease gene lists of four different publications released within the last year, the number of disease associated genes varied between 289 and 384: (1) Frazier et al. (2019), 289 genes, (2) Rahman (2020), 384 genes, (3) updated list of Stenton and Prokisch (2020), 343 genes, and (4) Falk (2020), 313 genes. Within these four lists, a total of 413 distinct mitochondrial disease associated genes were reported. Amongst these 413 genes, just 272 genes were included by all authors (Figure 1), illustrating their diversity. The following could be the reasons for the differences in the classification.

**FIGURE 2:**
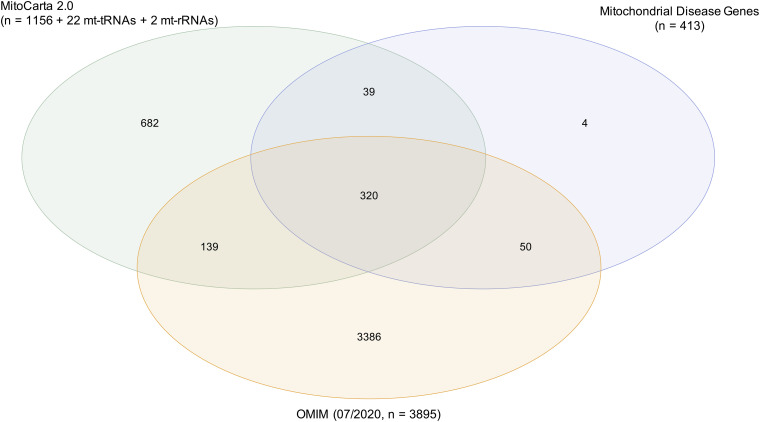
Characterization of mitochondrial disease genes. Mitochondrial localization of encoded proteins was examined by using the mitochondrial proteome inventory MitoCarta2.0 (1156 genes). The 22 mitochondrial tRNAs and 2 mitochondrial rRNAs were also considered as mitochondrially localized. A total of 87% (359/413 genes) of mitochondrial disease genes were located in the mitochondrion. Annotation in the database OMIM (07/2020) served as prove for the pathogenicity of genes. A total of 90% (370/413 genes) of mitochondrial disease genes were annotated in OMIM with an associated phenotype and mode of inheritance.

Firstly, the existing biochemical definition, that a mitochondrial disease must lead to direct OXPHOS defects, implies that the encoded protein is generally localized in the mitochondrion with very few exceptions. Using MitoCarta2.0, an inventory of the mitochondrial proteome, to examine the mitochondrial localization, 13% (54/413 genes) of the proteins encoded by the named genes are not listed (Figure 2). Though the two mitochondrial rRNAs and 22 mitochondrial tRNAs are not mentioned in MitoCarta2.0 due to their non-protein-coding properties, they also have a mitochondrial localization. No standardized definitions exist on whether gene products have to be located within the mitochondrion, on the mitochondrial membrane and responsible for the mitochondrial transport, or outside the mitochondrion and responsible for the mitochondrial metabolism, such as *RRM2B* (Bourdon et al., 2007; Chen et al., 2019). For instance, defects in the cytosolic tRNA synthetase *IARS*, necessary for the synthesis of nuclear-encoded mitochondrial proteins, are not regarded as a cause of primary mitochondrial disease, despite the fact of being associated with an OXPHOS defect (Kopajtich et al., 2016). Additionally, knowledge of mitochondrial proteins listed in MitoCarta2.0 inventory (released in 2016) is increasing over time, representing one reason why some proteins with a mitochondrial localization are not yet listed. To name an example, *TRMT5* was reported concurrently (Powell et al., 2015), making regular updates essential and to our knowledge currently in preparation.

Importantly, though defects in all 413 genes have been ascribed in the literature as associated with mitochondrial disorders, 43 (10%) genes are not yet annotated in OMIM^1^ as disease genes with an associated phenotype and mode of inheritance (status 07/2020) (Figure 2). Differences in the published mitochondrial disease gene lists and OMIM could be due to difficulties in assessing the evidence for inclusion and exclusion criteria. However, the evidence criteria to be fulfilled for annotation in OMIM are to our knowledge not consistent and their absence in OMIM is likely due to a work overload. Typically, description of a single case is not sufficient to define a genotype-phenotype association, but this seems not to be the limitation for the inclusion into OMIM, as for example in *TXN2* (Holzerova et al., 2016). As most knowledge databases, OMIM is dynamic and though regularly updated there may be a time-lag in inclusion, so that recently discovered disease genes may not yet be annotated in OMIM. References with evidence of pathogenicity were found in the literature for 40 of 43 mitochondrial disease genes not yet annotated in OMIM (Supplementary Table 1).

Taken together, although the biochemical definition of mitochondrial disorders relies on a defective OXPHOS, no gold standards have been developed for the classification of mitochondrial diseases. The annotation of proteins and disorders in MitoCarta2.0 and OMIM may contribute to the differences in the published disease gene lists.

**FIGURE 3:**
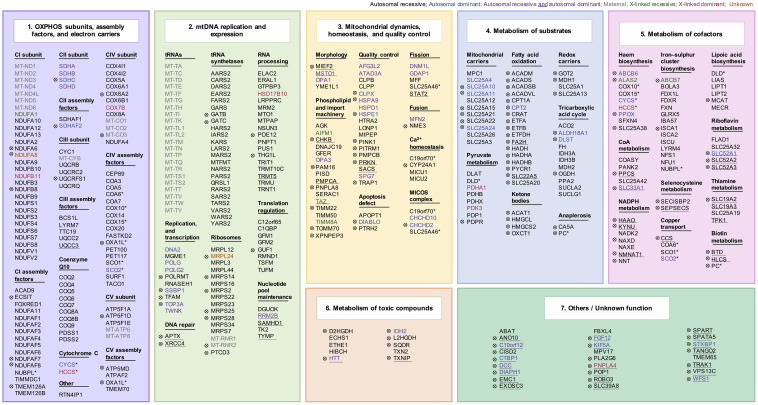
Mitochondrial disease genes. An association with mitochondrial disorders has been reported for 413 genes. Genes were grouped into subcategories according to their function: (1) OXPHOS subunits, assembly factors, and electron carriers (112/413 genes), (2) mitochondrial DNA replication and expression (104/413 genes), (3) mitochondrial dynamics, homeostasis, and quality control (55/413 genes), (4) metabolism of substrates (57/413 genes), (5) metabolism of cofactors (49/413 genes), (6) metabolism of toxic compounds (10/413 genes), and (7) others (26/413 genes). The mode of inheritance is indicated by color. A total of 13 genes have more than one mitochondrial function, indicated by an asterisk. Genes not included in the core set of all four publications (141/413 genes) are indicated by a sign (⊗). Genes encoding proteins not localized to the mitochondrion according to MitoCarta2.0 are highlighted with an underscore (54/413 genes).

Secondly, with increasing knowledge of the molecular, biochemical, and clinical basis of diseases, a clear definition of mitochondrial disorders becomes more complex. A wide array of proteins with a multitude of functional roles influence the functionality of the OXPHOS, impeding the biochemical definition and leading to an accelerated implementation of the genetic definition (Figure 3).

**FIGURE 4:**
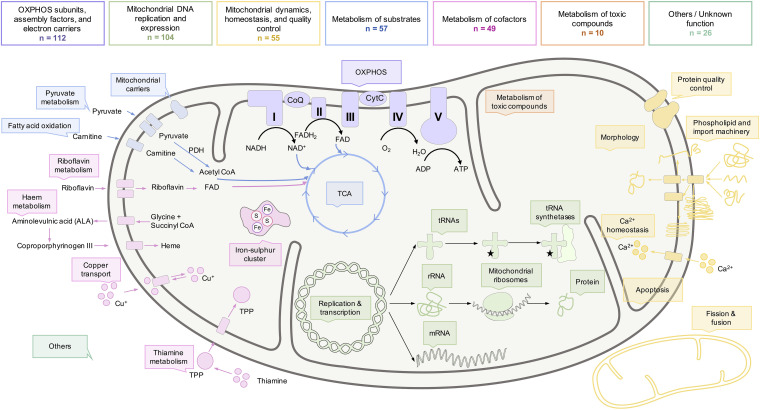
Functional roles of mitochondria and the biochemical processes within a mitochondrion. Although the OXPHOS is considered as the hallmark of mitochondria, multiple proteins with a wide range of functional roles influence the metabolism of mitochondria. Some of the functions and biochemical processes of mitochondria, commonly defective in mitochondrial diseases, are illustrated here. The number of mitochondrial disease genes implicated in the biochemical pathways is indicated. Genes with multiple functions were only listed within one category.

According to the functional roles of the encoded proteins, mitochondrial disease genes can be divided into seven subsets: (1) OXPHOS subunits, assembly factors, and electron carriers (112/413 genes), (2) mitochondrial DNA replication and expression (104/413 genes), (3) mitochondrial dynamics, homeostasis, and quality control (55/413 genes), (4) metabolism of substrates (57/413 genes), (5) metabolism of cofactors (49/413 genes), (6) metabolism of toxic compounds (10/413 genes), and (7) others/unknown function (26/413 genes) (Figure 4; Supplementary Table 2). Novel diseases associated with genes functionally assigned to the first or second subcategory of mitochondrial disease genes can be classified as mitochondrial diseases, as already accepted for pathogenic mtDNA variants. A mitochondrial localization of the encoded proteins is most likely. Genes within these functional categories not yet associated with mitochondriopathies represent strong prospective candidates. Proteins of the third category, mitochondrial dynamics, homeostasis, or quality control have generic mitochondrial functions, whose malfunction can directly or indirectly impair the mitochondrial energy metabolism. Diseases associated with a direct impairment of these functions are also commonly considered as mitochondriopathy. However, genetic defects in *PINK* or *PRKN*, which affect the mitochondrion but represent a specific subgroup of the disease categorized as Parkinson disease are an exception (Shimura et al., 2000; Valente et al., 2004). Besides the energy production via the OXPHOS, other aerobic energy metabolisms, such as the TCA cycle, pyruvate metabolism, or fatty acid oxidation, are part of the mitochondrial function and this metabolism of substrates required for the OXPHOS represents category four. Diseases associated with genes involved in the TCA cycle or the pyruvate metabolism are commonly considered as mitochondrial disorders. Defective PDH function is a prime example for not causing a classic OXPHOS dysfunction but being accepted as mitochondrial disease due to a defective oxidative decarboxylation of pyruvate (Brown et al., 1994). In contrast, functional subgroups of the mitochondrial substrate metabolism, such as fatty acid oxidation, ketone bodies, and anaplerosis, are controversially disputed as causative factors of mitochondriopathies as pathogenic mutations affect the mitochondrial energy metabolism but usually do not cause an OXPHOS deficiency. The OXPHOS requires at least ten cofactors (category 5). Dysfunctional OXPHOS due to cofactor deficiencies is generally regarded as a cause of mitochondriopathies. The association of a gene with a mitochondrial disorder is debated if the encoded protein is not located to the mitochondrion, such as with riboflavin transporters in the plasma membrane. Nevertheless, defects of the riboflavin transporters finally entail a lack of riboflavin in the mitochondrion and an associated impairment of mitochondrial function (Udhayabanu et al., 2017). In addition, certain mitochondrial diseases can arise from toxic metabolites that attack the respiratory chain. The latter group (category 7) comprises mainly proteins with a function outside the mitochondrion or a function not yet fully known. Disruptions in their function can, however, indirectly provoke mitochondrial dysfunctions posing the question as to what the main effect of a gene is.

The pathways currently reported in the context of mitochondrial disorders are presumably not yet complete and ongoing research in the field will continue to expand this list, just as knowledge about the localization and function of proteins is equally dynamic. Given the dynamic nature of mitochondrial disease genes, genes such as *MRPL24* (Di Nottia et al., 2020) may not yet be annotated in OMIM or included in all scientific publications. Furthermore, we are constantly discovering new factors involved in mitochondrial function further enriching the list of mitochondrial disease associated genes.

Taken together, the high level of clinical and genetical complexity, in combination with the absence of reliable biomarkers, resulted in the unavailability of a precise biochemical and genetic definition of mitochondrial disorders. No uniform and standardized definitions, regarding the pathogenicity, the presence of an OXPHOS defect, and the mitochondrial localization, have been provided to classify a gene or variant as a cause of mitochondriopathies (Haas et al., 2008). Moreover, diagnosis of mitochondrial disorders is increasing based on gene function, and a biochemical defect cannot be detected in all patients with pathogenic variants, further complicating the definition of mitochondrial diseases. All these aspects for the inclusion and exclusion of mitochondrial disease genes can be assessed differently, and consequently lead to different gene lists, ranging from 270 to over 400 disease genes.

## Future Perspectives

With steadily growing utilization and further advancement of sequencing methodologies, the number of pathogenic variants and genes associated with mitochondrial disease will constantly rise. Raising the question of whether a precise definition of mitochondrial disease genes is indeed useful, and whether it is of importance to speak of 270 or 400 genes.

For patients with a constellation of symptoms indicating mitochondrial dysfunction in the absence of clear pathogenic variants and biochemical defects, it is recommended to avoid the diagnostic term “possible” mitochondrial disease (Parikh et al., 2019). Instead, in patients remaining without diagnosis, the focus should be on the description of the clinical manifestation. In fact, with regard to the rarity of mitochondrial diseases, the suspicion of a mitochondrial disorder should qualify for the inclusion into mitochondrial patient registries. Through the acquisition of patient data, patient registers are a powerful tool whose analytical statistical significance increases with a growing number of patients included. As research on such registries is ongoing, the chances of finding a molecular diagnosis or assessing the effectiveness of therapies will improve in the future. For patients suspected of having a mitochondrial disease, inclusion into patient registries not only provides diagnostic advantages, but also enables psychological support for the patients. The collaboration of clinicians and researchers facilitates an exchange of data worldwide, accelerates gene discoveries, and improves our understanding of genotype-phenotype correlations. This provides the opportunity to coordinate sufficiently powered clinical trials (Schon et al., 2020). The emergence of national mitochondrial patient registers over the last few years (e.g., mitoNET, MITOCON, NAMDC) has not only enabled large-scale NGS studies to be conducted to further clarify genotype-phenotype correlation (Altmann et al., 2016). It also offers other benefits such as in conducting natural history studies, in the preparation of intervention studies, the establishment of biobanks, and in increasing public and clinical awareness. Meanwhile, GENOMIT, a global mitochondrial patient registry with eight partners, in Germany, Austria, France, Italy, United Kingdom, Japan, and the United States, has been established. This global exchange of information will enable tremendous progress in this complex disease.

## Conclusion

Mitochondrial disorders are associated with an increasingly large number of heterogenous clinical and molecular presentations. By moving further into the genomic and multi-omic diagnostic era, the diagnosis of a mitochondrial disease on the basis of conventional biochemical definitions is increasingly being challenged. The function of mitochondria in many cellular activities and pathways contributes to the complexity in defining a single list of mitochondrial disease genes. Therefore, instead of adhering to an overly precise disease definition, the generous inclusion of patients with suspected mitochondrial disease, based on clinical and genetic evidence, into global patient registries appears to be more practicable and also beneficial to the field. The enlargement of global patient registries not only permits psychological support for patients, but also helps to combine clinical and genetic data in order to be able to analyze disease pathomechanisms in greater detail. Within the framework of the associated research studies, the establishment of genetic and biochemical criteria for the specific definition of mitochondrial diseases lends itself to the formation of clear patient groups. Moreover, advancements in diagnostic techniques are further unraveling the underlying causes of mitochondrial disease and will hopefully pave the way for the development of powerful treatments or curative agents for patients.

## Author Contributions

This article was written by LS under the guidance of HP.

## Conflict of Interest

The authors declare that the research was conducted in the absence of any commercial or financial relationships that could be construed as a potential conflict of interest.

## Footnotes

1 Online Mendelian Inheritance in Man, OMIM^®^. McKusick-Nathans Institute of Genetic Medicine, Johns Hopkins University (Baltimore, MD). https://omim.org/ (accessed July 15, 2020).

## Supplementary Material

Supplementary Table 1 Evidence for pathogenicity for 45 published mitochondrial disease genes not listed in OMIM.

Supplementary Table 2 Current list of published mitochondrial disease genes.

## Abbreviations

ACAD9: Acyl-CoA dehydrogenase family member 9
ATP: adenosine triphosphate
CPEO: chronic progressive external ophthalmoplegia
FA: Friedreich ataxia
KSS: Kearns-Sayre syndrome
LHON: Leber’s hereditary optic neuropathy
MELAS, mitochondrial encephalopathy: lactic acidosis and stroke-like episodes
MIDD: maternally inherited diabetes and deafness
mtDNA: mitochondrial DNA
MT-ND4: mitochondrially encoded NADH:ubiquinone oxidoreductase
MT-RNR2: mitochondrially encoded 16S rRNA
MT-TL1: mitochondrially encoded tRNA leucine 1
nDNA: nuclear DNA
NGS: next generation sequencing
OMIM: Online Mendelian Inheritance in Man
OXPHOS: oxidative phosphorylation
PDHA1: pyruvate dehydrogenase E1 subunit alpha 1
RNA-seq: RNA sequencing
TCA cycle: tricarboxylic acid cycle
tRNA: transfer RNA
VUS: variant of unknown significance
WES: whole-exome-sequencing
WGS: whole-genome-sequencing.
